# Artificial intelligence in child nutrition and eating behavior: from prediction to gastronomic mediation

**DOI:** 10.3389/fnut.2026.1952364

**Published:** 2026-08-24

**Authors:** Lu Xing, Xueqi Wu

**Affiliations:** 1 School of Preschool Education, Changsha Normal University, Changsha, China; 2 School of Education, Faculty of Social Sciences and Leisure Management, Taylor’s University, Subang Jaya, Malaysia; 3 Taylor’s Culinary Institute, Faculty of Social Sciences and Leisure Management, Taylor’s University, Subang Jaya, Malaysia

**Keywords:** artificial intelligence, child nutrition, child-centered design, digital food environment, eating behavior, food culture, gastronomy, personalized nutrition

## Abstract

Artificial intelligence (AI) is entering child nutrition through dietary assessment, malnutrition forecasting, clinical decision support, meal recommendation, conversational interventions, and food-environment monitoring. The consequences of these applications converge in everyday eating. This Mini Review synthesizes evidence on how AI measures nutritional states and mediates food choice, communication, sensory acceptance, family practice, and digital exposure. The evidence supports two connected functions. As nutritional intelligence, AI converts clinical, dietary, and environmental data into assessments or predictions. As gastronomic mediation, it participates in decisions about what foods are noticed, recommended, prepared, discussed, and accepted. Direct pediatric validation is strongest for bounded assessment and forecasting tasks. Child meal-planning studies and generative-AI evaluations based on standardized adolescent profiles reveal a gap between nutrient optimization, culinary coherence, and nutritional safety. A large adolescent chatbot trial combined scalable delivery with null intention-to-treat effects on diet quality and BMI trajectory. Co-design and behavioral studies further identify familiarity, texture, participation, and caregiver involvement as central design variables. We propose five iterative translational gates: technical validity, nutritional validity, behavioral acceptability, contextual legitimacy, and real-world effectiveness and implementation. Future research should combine age-specific nutritional constraints with sensory and cultural knowledge, evaluate performance across food cultures, preserve professional and caregiver oversight, and test outcomes in homes, schools, clinics, and digital food environments. AI can advance child-focused gastronomy by translating computational outputs into safe, culturally meaningful, and developmentally appropriate eating practices.

## 1 Introduction

Child nutrition integrates nutrient supply with household resources, health conditions, feeding relationships, food availability, and the cultural organization of meals. The continuing coexistence of undernutrition, micronutrient deficiency, food poverty, and excess weight makes this complexity visible at a global scale (1, 2). For children, dietary decisions are also relational: caregivers, peers, schools, media, and commercial platforms shape what can be eaten, what is considered desirable, and which foods become familiar.

Artificial intelligence (AI) is increasingly positioned as a way to manage this complexity. In pediatric nutrition, machine-learning systems classify malnutrition risk, support clinical decisions, analyze dietary images, forecast population-level vulnerability, personalize meal plans, and deliver behavior-change messages (3, 4). Here, AI includes statistical machine learning, knowledge- and rule-based systems, optimization, computer vision, structured conversational agents, and generative models, each with distinct risks and evidence standards. Recent applications include image-assisted dietary assessment and food-exposure measurement (5, 6), a large adolescent chatbot trial (7), child and family meal-planning systems (8, 9), and direct tests of AI-generated school meals and adolescent diet plans (10, 11).

The field still prioritizes model performance over the eating situation into which a system intervenes. An AI-generated school meal was rejected in one comparison because its texture or flavor was unfamiliar (10). A fluent recommendation may also be nutritionally unsafe (11), unaffordable, unavailable, religiously inappropriate, or incompatible with shared family meals. A conversational agent can increase engagement while narrowing autonomy or transmitting stereotypes. Computer vision can reduce recording burden, yet error profiles for mixed dishes and underrepresented cuisines remain poorly characterized. These characteristics determine whether AI improves nutrition or produces plausible outputs without practical value, and they require explicit governance (12).

This distinction is especially important for the future of gastronomy. Gastronomy joins nutritional composition to sensory experience, culinary practice, memory, identity, social relationships, and food environments. Child-facing AI enters this domain whenever it recognizes a food, recommends a meal, frames a health message, assists a caregiver, or monitors persuasive digital content. It therefore acts both as nutritional intelligence—infrastructure for measuring and predicting nutritional states—and as gastronomic mediation—a participant in the processes through which foods are selected, prepared, represented, and experienced.

This Mini Review advances a two-function account of child-nutrition AI centered on the translation of computational output into eating practice. It examines what current applications have demonstrated, where nutritional and gastronomic evidence diverge, and which standards should govern child-facing translation. Culture, cognition, and equity define the conditions under which AI becomes meaningful for children, families, clinicians, and food systems. The implications are developmentally patterned: caregiver-mediated sensory learning is especially important in infancy and the preschool years; school-aged children increasingly participate in preparation and choice; and adolescents exercise greater food autonomy while encountering more direct digital exposure.

## 2 Review approach

This focused Mini Review synthesizes peer-reviewed studies and authoritative guidance published through 29 July 2026. A targeted search of Web of Science, Scopus, and PubMed was combined with reference tracing from recent pediatric nutrition reviews and updated searches for validation studies. The search combined AI terms (“artificial intelligence,” “machine learning,” “deep learning,” “computer vision,” chatbot, “large language model,” or “generative AI”) with pediatric terms (child, adolescent, pediatric/paediatric, infant, or preschool) and nutrition terms (nutrition, diet, feeding, eating behavior, dietary assessment, meal planning, food choice, food environment, or food marketing). Selection was restricted to English-language publications, with no geographic restriction. Titles and abstracts were screened for relevance, followed by full-text assessment of potentially eligible records. Direct evidence was eligible when an implemented AI system addressed nutrition, feeding, eating behavior, or food environments for people younger than 18 years, their families, or child-facing settings. Reviews, guidance, and indirect studies were retained only for mechanisms, design, or governance; records without substantive child-nutrition relevance or a defined evidentiary role were excluded. Development, validation, intervention, and co-design studies were examined for established findings and priority evidence gaps (3, 4). World Health Organization guidance informed the interpretation of governance issues (12).

Evidence was interpreted according to study design. Participant-based pediatric AI studies supported claims about child or adolescent performance and outcomes. Studies using pediatric profiles without pediatric participants were treated as pediatric-targeted technical evidence. Adult AI experiments and pediatric digital, participatory, or behavioral studies without substantive AI components were treated as indirect or supportive evidence for mechanisms, design, and validation requirements. The synthesis addressed two questions: whether an AI system produces a technically and nutritionally credible output, and whether that output functions within children’s sensory, behavioral, familial, cultural, and institutional contexts.

## 3 Thematic findings

### 3.1 Nutritional intelligence: prediction, surveillance, and clinical support

Frequently represented applications in the selected literature treat AI as an analytical layer over structured clinical, survey, or environmental data. Machine-learning models have predicted under-five malnutrition from household and demographic variables (13), forecast acute malnutrition from high-frequency monitoring (14), and incorporated environmental or conflict conditions as leading indicators (15). Related studies classify stunting or use machine learning within causal analyzes of health-service exposure and nutritional outcomes (16, 17). These applications could support prioritization of scarce resources toward high-risk places or patients, particularly where repeated anthropometry and specialist capacity are limited.

Their operational value lies in prioritization. High-frequency forecasting may shorten the interval between deteriorating conditions and humanitarian response (14). Prediction gains value when linked to an actionable threshold, a responsible institution, and an intervention capable of changing the trajectory. A model trained on one survey architecture, conflict pattern, or referral population may encode relationships that shift across regions or time.

Clinical applications present a similar pattern. A machine-learning model developed from maternity-ward data predicts exclusive breastfeeding (18). Its clinical value now depends on calibration, external validation, integration with clinical history and nutritional standards, responsibility for overrides, and consequences for families. These criteria connect predictive discrimination to precision in care.

### 3.2 From dietary measurement to meal recommendation

Dietary assessment is a pivotal bridge between nutritional intelligence and everyday eating. Conventional self-report is burdensome and vulnerable to omissions, portion-size error, and reactivity, particularly in young people (19). Mobile image capture and AI-assisted recognition can reduce this burden. In a relative-validity study of 36 Vietnamese girls aged 12–18 years, the FRANI application was equivalent to weighed records within a 10% bound for energy, protein, fat, and four micronutrients; concordance correlation coefficients for energy and most nutrients ranged from 0.60 to 0.81, while omission and intrusion rates were each approximately 21% (5). These results show both utility and residual error. Wearable cameras combined with deep learning have also been used to capture children’s food exposure (6), extending measurement beyond intake alone.

These systems nevertheless inherit a difficult recognition problem. Children’s meals may be shared, partially eaten, combined, or prepared as mixed dishes whose ingredients are not visually separable. Food appearance changes with cooking, serving style, and local convention. An image classifier trained on standardized or commercially common items may perform less reliably for household dishes or culturally specific foods. Portion estimation adds another layer of uncertainty. Consequently, a convenient measurement system can create a misleading sense of precision unless errors are reported by food type, preparation, age, and cultural context.

Meal planning moves from observation to prescription. Lee et al. trained generative-adversarial and reinforcement-learning systems using 1,726 food items and 220 daily plans for children aged 3–5 years (8). Experts rated the reinforcement-learning plans favorably when only nutrient information was visible, while human-designed diets were viewed more positively when food names revealed the actual compositions. The divergence exposes a gap between nutrient compliance and meal coherence. The family-oriented SWITCHtoHEALTHY system generated plans from 32 family profiles. Children’s daily-rule compliance averaged 96% under preferred rules and 99% under less restrictive rules, whereas weekly compliance rose from 71 to 91% after rule relaxation (9). These results establish technical rule compliance and define the next outcomes for evaluation: intake, acceptance, and persistence.

Generative AI intensifies both the opportunity and the risk. Bilen et al. compared 60 three-day plans produced by five general-purpose AI models with dietitian plans for standardized adolescent profiles (11). The AI plans underestimated energy by a mean of approximately 695 ± 388 kcal (Cohen’s *d* = 1.79), produced carbohydrate shares below recommended ranges, and varied substantially in micronutrient content. An adult proof-of-principle study, used here only as indirect design evidence, reported that blinded sensory evaluation with 101 participants showed how explicitly constrained models can explore trade-offs among taste, nutrition, and sustainability (20). This adult finding does not establish pediatric acceptability or effectiveness; it illustrates a candidate design strategy for subsequent child-specific testing. Together, the pediatric-targeted comparison based on standardized adolescent profiles and the indirect adult experiment motivate pediatric design requirements: constraint-based nutrient calculation, verified composition data, allergy and safety rules, culinary checks, and professional oversight.

### 3.3 Gastronomic mediation: acceptance, participation, and digital choice architecture

AI influences nutrition through both what it recommends and how children encounter food. In a randomized trial of 7,890 adolescents, approximately 63% of those allocated to an SMS chatbot program joined it, demonstrating scalable delivery and moderate uptake (7). Intention-to-treat analyzes found no improvement in diet quality or BMI trajectory. A small decrease in BMI *z*-score at 6 months appeared in a per-protocol analysis, without a significant diet-quality effect. The trial separates delivery from effectiveness: reach, engagement, and personalization alone did not change the primary outcomes. Its structured SMS design also shows why technology class matters when interpreting conversational interventions.

Participation changes the quality of mediation. Co-design research on a positive body-image chatbot with young people and parents revealed concerns that developers may overlook (21). School-meal evidence makes the contrast even clearer. Goulart et al. compared carrot-peel pies proposed by children, school cooks, and ChatGPT (10). Children accepted the recipes created by themselves and the cooks, whereas the AI-generated recipe was rejected and evoked negative emotions, with unfamiliar taste and texture among the explanations. As indirect pediatric support rather than direct AI evidence, home-cooking feasibility research contributes measures of preparation, repeated exposure, and family participation (22). Separate non-AI evidence in infants and toddlers indicates that repeated tasting across approximately 8–10 or more exposures can increase acceptance of exposed fruits or vegetables (23). These studies inform candidate behavioral mechanisms and outcome measures; they do not establish the effectiveness of an AI intervention. Together with the direct AI comparison, they identify sensory learning, agency, familiarity, and embodied experience as core variables for child-facing systems.

The digital food environment is a second site of gastronomic mediation. A meta-analysis of 80 articles and 19,372 participants associated food marketing with increased intake, choice, and preference in children and adolescents, although certainty ranged from very low to moderate (24). World Health Organization guidance consequently treats food marketing as a child-rights and nutrition concern (25). AI can help detect unhealthy promotion at a scale that may exceed feasible manual monitoring, including branded content embedded in social media, games, and influencer posts (26). The same computational capacities also support microtargeting, persuasive optimization, and individualized promotion. AI’s effect follows the objective function: public-health monitoring seeks to reduce harmful exposure, whereas commercial systems may optimize attention and purchase behavior. Governance must address both uses.

Three recurring patterns emerged across the selected studies and are summarized in Table 1. First, the most interpretable validation occurs when the task is bounded and the reference standard is explicit. Second, the transition from prediction to recommendation increases the need for nutritional, sensory, and contextual validation. Third, participatory evidence contributes information beyond nutrients, clicks, and images.

**Table 1:** Evidence and translational priorities for AI in child nutrition and eating behavior.

Application domain	Representative evidence and finding	Current contribution	Priority for translation
Risk modeling and early warning	Retrospective survey models (13, 16, 17); high-frequency and contextual forecasting (14, 15).	Models estimate or stratify risk and support anticipatory prioritization.	Prospective calibration, action thresholds, institutional response, and fairness across settings.
Clinical feeding and decision support	Breastfeeding prediction-model development (18); focused pediatric review (3).	Models identify clinically relevant risk for professional review.	External validation, workflow impact, family-centered explanation, and accountability for overrides.
Dietary assessment and food-exposure measurement	Relative-validity study, *n* = 36 (5); observational wearable-camera study (6).	FRANI quantified nutrient agreement and residual error; cameras extended measurement to visual food exposure.	Error reporting for mixed dishes, portion size, age groups, and diverse food cultures.
Meal planning and personalized recommendation	Expert appraisal (8); technical rule-compliance validation (9); 60-plan comparative nutritional validation (11).	Nutrient and rule compliance diverged from culinary coherence, child acceptance, and safety.	Verified nutrient engines, sensory testing, affordability, allergies, family routines, and professional oversight.
Conversational and behavioral support	Randomized trial, *n* = 7,890 (7); participatory chatbot co-design (21).	Delivery scaled, intention-to-treat dietary and BMI effects were null, and co-design defined acceptability requirements.	Mechanism-specific outcomes, durable effects, autonomy, developmental appropriateness, and participatory design.
Culinary co-creation and food acceptance	Direct pediatric AI sensory comparison (10); indirect/supportive cooking-feasibility (22) and repeated-exposure evidence (23).	Familiarity, texture, agency, and repeated exposure shaped acceptance.	Repeated real-world testing across cuisines, ages, households, and school systems.
Digital food-environment monitoring	Marketing meta-analysis (24), authoritative guidance (25), and AI-focused policy perspective (26).	Marketing shapes eating-related outcomes, while AI extends monitoring and persuasive targeting.	Transparent detection, cross-language performance, enforceable platform governance, and limits on persuasive AI.

## 4 Discussion

### 4.1 A two-layer account of child-nutrition AI

We interpret the evidence through two interacting layers. Nutritional intelligence converts data into estimates of intake, risk, response, or nutritional adequacy. Gastronomic mediation converts outputs into encounters with foods: a recommendation appears on a screen, a caregiver receives an alert, a meal is assembled, a child experiences a texture, or a platform amplifies an advertisement. The first layer asks whether the output is analytically correct. The second asks whether it changes eating in a way that is acceptable, meaningful, and beneficial.

Neither layer is sufficient alone. A culturally appealing recommendation that fails age-specific nutrient requirements is unsafe. A nutritionally correct recommendation that ignores taste, food skills, cost, religion, family schedules, or local availability is inert. The interface between the layers is therefore the decisive research object. It is where technical performance becomes—or fails to become—nutritional value.

Existing clinical-AI guidance such as DECIDE-AI emphasizes early prospective evaluation, safety, human factors, and workflow integration (27). Our model retains those requirements and adds a gastronomic mediation layer: a computational output must become a sensory and socially situated food encounter before it can affect nutrition. Culinary coherence, age-dependent food agency, cultural legitimacy, and digital persuasion therefore become explicit translational gates. This child-specific extension defines a prospective research agenda for child-nutrition AI.

**Figure 1:**
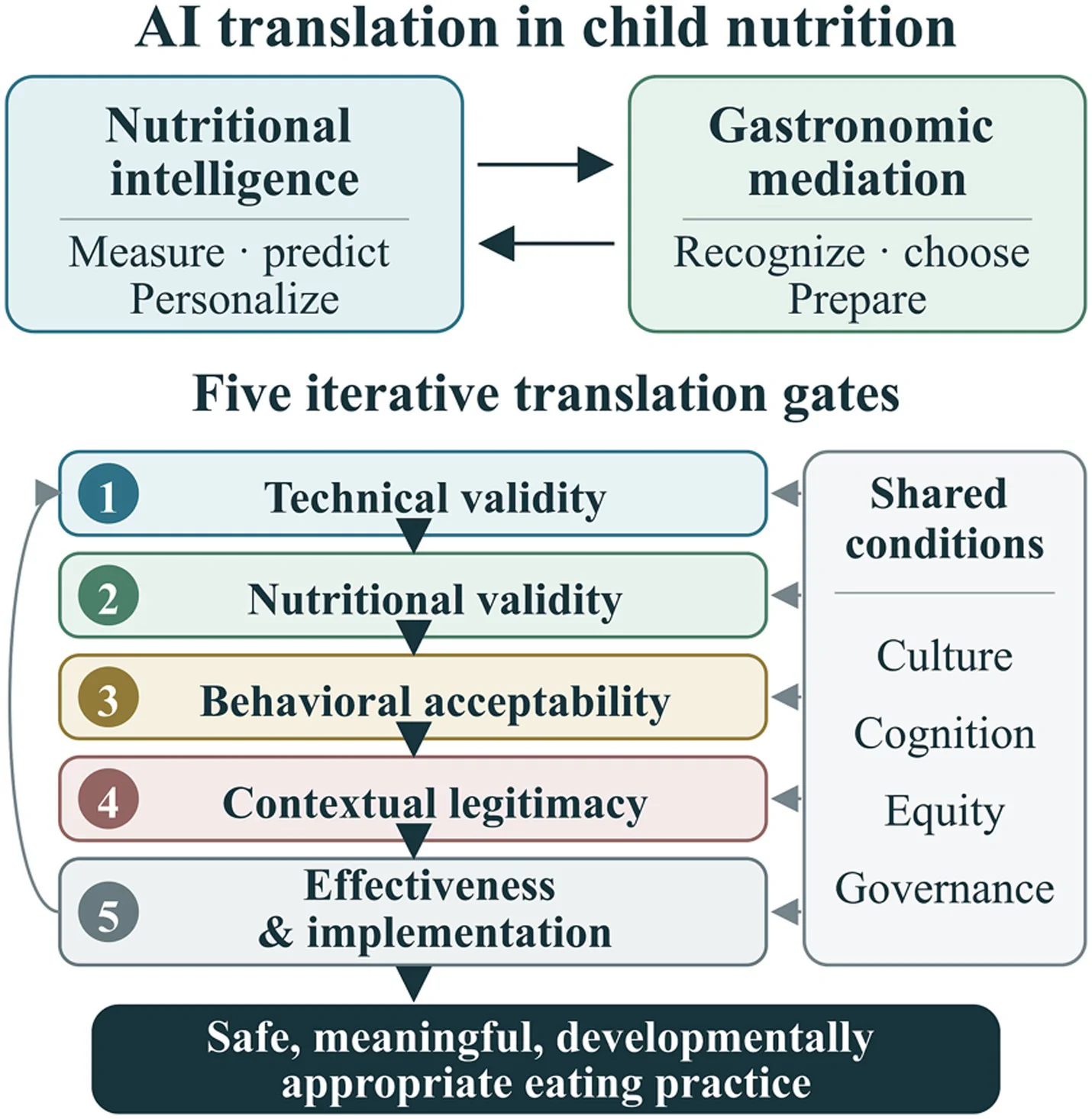
Two-layer framework for translating child-nutrition AI into gastronomic practice.

Figure 1 formalizes this relationship. Culture, cognition, equity, and governance operate as shared conditions across the full pathway. In this proposed framework, culture concerns whether ingredients, preparation methods, and meal structures are represented and considered legitimate. Cognition concerns attention, learning, and developing autonomy in food exposure and persuasive environments (23, 24). Equity concerns who is represented in data, who can access a system, and who bears its errors or burdens. Governance concerns oversight, privacy, contestability, commercial incentives, and responsibility when recommendations cause harm.

### 4.2 Translation requires five iterative gates

The synthesis suggests five gates. Technical validity can be assessed through calibration, discrimination, error by food or subgroup, and external performance. Nutritional validity requires age-specific energy and nutrient adequacy, growth and allergy safeguards, and agreement with verified composition data. Behavioral acceptability requires sensory acceptance, comprehension, usability, autonomy, and absence of stigmatizing or restrictive effects. Contextual legitimacy requires fit with food culture, affordability, availability, household routines, and institutional capacity. Real-world effectiveness and implementation require sustained dietary or clinical benefit, reach, fidelity, workload, unintended effects, and distributional outcomes.

Most studies selected for this Mini Review evaluated the first gate and some addressed the second. The evidence from child diet planning and adolescent generative-AI diets shows why they must be separated (8, 11). One system may satisfy nutrient targets while proposing an implausible composition; another may produce a coherent-looking menu while underestimating energy and micronutrients. Sensory and participatory studies then show that nutritional and behavioral evidence can also diverge (10, 22, 23). Feedback makes the sequence iterative: rejection, unaffordability, workload, or an adverse outcome returns the system to earlier design and validation steps.

This framework also changes what counts as validation. For dietary image analysis, aggregate accuracy should be accompanied by errors for mixed dishes, household preparations, and underrepresented foods. For malnutrition forecasting, calibration should be paired with evidence that alerts reach institutions able to respond. For conversational agents, use frequency should be complemented by dietary, psychosocial, and autonomy outcomes: the large chatbot trial shows that reach can coexist with null intention-to-treat effects (7). For meal recommenders, offline nutrient checks should precede sensory testing, household feasibility, and longer-term dietary evaluation.

### 4.3 Culture, cognition, and equity are model inputs, outcomes, and safeguards

Our synthesis specifies three measurable levels of cultural fit. Representational fit asks whether local dishes, ingredients, languages, and preparation methods are present and correctly encoded; studies can report coverage and error by cuisine or dish type. Practical fit asks whether recommendations suit budgets, equipment, shopping patterns, school meals, and household time; studies can measure cost, availability, preparation burden, and adherence. Relational legitimacy asks whether children, caregivers, cooks, dietitians, and communities recognize the system as an acceptable participant in food decisions; qualitative and participatory methods are needed. These domains turn cross-cultural validation into a measurable research program.

These levels can conflict. A recommender may recognize a regional dish but propose it at an unaffordable frequency. A chatbot may speak the correct language while using an adult, moralizing, or stigmatizing tone. A system may personalize to a child’s prior choices so closely that it reinforces a narrow diet and reduces exposure to new foods. Cultural adaptation therefore requires observation and participation beyond surface localization.

Cognition likewise has two roles. It is an intervention target when systems seek to change knowledge, attention, motivation, self-monitoring, or food choice. It is also a proposed safety domain because developmental capacity and decision authority differ markedly by age. For infants and preschool children, sensory learning, texture, repeated exposure, and caregiver mediation dominate. School-aged children increasingly participate in preparation and choice but still require strong caregiver and school safeguards. Adolescents have greater autonomy and direct platform exposure, making transparent persuasion, body image, restrictive eating, and privacy especially salient. Systems should therefore disclose why a recommendation appears, preserve meaningful alternatives, avoid manipulative reward structures, and enable caregiver or professional review appropriate to age and risk.

Equity should be evaluated across access, representation, performance, and burden. Device ownership, connectivity, data costs, language availability, digital literacy, caregiver time, food prices, and local availability can determine who can use a system and act on its outputs. Training data that underrepresent low-resource settings, minority languages, local dishes, disabilities, or diverse body sizes may produce differential recognition, prediction, or recommendation errors. Studies should therefore report access barriers and disaggregate performance and implementation burden by socioeconomic position, language, geography, age, sex, and other contextually relevant groups, with participatory testing in populations most likely to be underserved (12).

### 4.4 Controversies and risk boundaries

Four controversies cut across the evidence. The first is personalization versus dietary diversity: adapting to prior preferences may improve short-term acceptability yet continually recommending familiar foods could narrow exposure and learning. The second is participation versus manipulation: interactive prompts can support agency, but the same techniques can optimize compliance without meaningful assent. The third is monitoring versus surveillance: image and platform data can reduce reporting burden or identify harmful marketing, while also capturing family members, peers, locations, brands, and routines beyond the nutritional purpose. The fourth is cultural adaptation versus standardization: standardized nutrient constraints protect safety, but standardized food ontologies can erase mixed dishes, local preparation, and shared-meal meanings. These tensions require governance criteria beyond model accuracy.

In this review, we propose three conceptual risk bands to align evaluation and oversight with potential harm; they are not an externally validated pediatric AI classification and do not replace clinical judgment. Within this proposed framework, lower-risk uses include general nutrition education and research-oriented food recording with child assent, caregiver permission appropriate to age, privacy protection, and non-stigmatizing design. Proposed medium-risk uses include meal suggestions or behavior prompts for otherwise healthy children; these require verified nutrient logic, caregiver controls, transparent recommendation reasons, and monitoring for restrictive or stigmatizing messages. Proposed high-risk uses include infant feeding, allergies, avoidant/restrictive food intake disorder, other eating disorders, swallowing or texture restrictions, metabolic disease, and medical nutrition therapy. For these uses, we propose clinician or dietitian authorization, deterministic safety rules, stop criteria, abnormal-intake alerts, auditable calculations, and human escalation. The approximate 695-kcal mean deficit observed in plans generated for standardized adolescent profiles supports professional supervision and auditable nutrient checks before child-facing use (11).

### 4.5 Research gaps and future development

Four priorities follow. First, evaluation should move from single-metric model studies to linked validation chains. A minimum study sequence would report external technical performance, nutrient adequacy against an age-specific reference, sensory or usability testing, household or institutional feasibility, and a sustained dietary or clinical outcome as distinct claims. Computational studies intended for use should include empirical validation with children, caregivers, professionals, or real food environments.

Second, research should build multimodal, culturally plural food knowledge. Food images, recipes, nutrient databases, preparation methods, price and availability data, language, and sensory descriptors must be linked without treating dominant commercial foods as universal. Dataset documentation should identify geographic and culinary coverage. Multisite studies should prospectively compare recognition error, recommendation feasibility, and acceptance across dishes, languages, ages, and socioeconomic conditions.

Third, AI should be designed as constrained collaboration. Nutrient calculators, allergy rules, clinical thresholds, and food-safety requirements should remain deterministic and auditable where possible. Generative systems can support explanation, variation, or co-creation, while dietitians, caregivers, school-food professionals, and children retain roles in setting goals and rejecting outputs. The school-meal comparison showed that child and cook involvement revealed sensory and contextual information absent from the AI-generated recipe (10).

Fourth, governance must cover the wider digital food system. Privacy, bias, explainability, and responsibility remain essential in clinical and household tools (12). In commercial environments, evaluation should disclose whether recommendation, advertising, and data monetization are controlled by the same actor. The same AI techniques that detect harmful promotion can optimize it, while marketing evidence shows measurable effects on children’s intake and choice (24). Child-nutrition policy must therefore evaluate objectives, data flows, incentives, and consequences behind every “AI for health” label.

### 4.6 Limitations of the evidence base and this review

The literature is heterogeneous in populations, data types, outcomes, and validation standards, which limits comparison across application streams. Many studies remain retrospective, single-setting, or technically focused. Evidence on cultural adaptation, long-term eating behavior, external validation, and implementation is thinner than evidence on model development. The targeted narrative design enables cross-domain conceptual integration but may introduce study-selection and emphasis bias; the English-language restriction and reliance on readily indexed literature may also have excluded relevant evidence. Several elements of the two-function model, five translational gates, and proposed risk bands arise from conceptual synthesis across direct, indirect, and supportive evidence rather than extensive direct pediatric validation. These propositions require prospective testing and refinement across developmental stages, low-resource settings, and diverse food cultures; future systematic reviews should add pooled effect estimation and formal risk-of-bias grading as outcomes become comparable.

## 5 Conclusion

AI in child nutrition is moving from measurement and prediction toward direct participation in food decisions. Published studies document promising, task-bounded roles in assessment, forecasting, and monitoring, but autonomous recommendation lacks adequate pediatric outcome evidence. Nutrient targets, fluent recommendations, scalable delivery, and high technical performance do not guarantee safe or accepted meals. The future of child-focused gastronomic AI depends on passing five iterative gates: technical validity, nutritional validity, behavioral acceptability, contextual legitimacy, and real-world effectiveness and implementation. Systems should be tested by age and risk, grounded in culturally plural food knowledge, and designed so that children, caregivers, cooks, and professionals retain meaningful authority. Under those conditions, AI may strengthen the human relationships through which children learn to eat.
